# Noninvasive Detection of Thin-Liquid Aspiration Using Dual-Axis Swallowing Accelerometry

**DOI:** 10.1007/s00455-012-9418-9

**Published:** 2012-07-28

**Authors:** Catriona M. Steele, Ervin Sejdić, Tom Chau

**Affiliations:** 1 Toronto Rehabilitation Institute, University Health Network, 550 University Avenue, #12-101, Toronto, ON M5G 2A2 Canada; 2 Bloorview Research Institute, Toronto, ON Canada; 3 Department of Speech-Language Pathology, University of Toronto, Toronto, ON Canada; 4 Institute of Biomaterials and Biomedical Engineering, University of Toronto, Toronto, ON Canada; 5 Department of Electrical Engineering, University of Pittsburgh, Pittsburgh, PA USA

**Keywords:** Deglutition, Swallowing, Dysphagia, Screening, Aspiration, Accelerometry, Deglutition disorders

## Abstract

Aspiration (the entry of foreign contents into the upper airway) is a serious concern for individuals with dysphagia and can lead to pneumonia. However, overt signs of aspiration, such as cough, are not always present, making noninstrumental diagnosis challenging. Valid, reliable tools for detecting aspiration during clinical screening and assessment are needed. In this study we investigated the validity of a noninvasive accelerometry signal-processing classifier for detecting aspiration. Dual-axis cervical accelerometry signals were collected from 40 adults on thin-liquid swallowing tasks during videofluoroscopic swallowing examinations. Signal-processing algorithms were used to remove known sources of artifact and a classifier was trained to identify signals associated with penetration-aspiration. Validity was measured in comparison to blinded ratings of penetration-aspiration from the concurrently recorded videofluoroscopies. On a bolus-by-bolus basis, the accelerometry classifier had a 10 % false-negative rate (90 % sensitivity) and a 23 % false-positive rate (77 % specificity) for detecting penetration-aspiration. We conclude that accelerometry can be used to support valid, reliable, and efficient detection of aspiration risk in patients with suspected dysphagia.

Prandial aspiration, or the entry of foreign material into the upper airway during swallowing, is a serious component of oropharyngeal dysphagia. Aspiration severity is usually subclassified according to the 8-point Penetration-Aspiration Scale [1], which scores severity according to the depth of airway invasion and the subsequent response observed during videofluoroscopic swallowing examinations. Normal airway protection receives a score of 1, while transient entry of material into the laryngeal vestibule (above the vocal cords) is termed *high penetration* and receives a score of 2. Scores of 3–5 (*penetration*) apply when material enters the laryngeal vestibule without subsequent clearance. *Aspiration* is the term used when material crosses the vocal cords and enters the trachea (scores of 6–8). A major dilemma for the detection of aspiration during clinical assessment is the fact that overt clinical signs, (e.g., cough or throat clearing), are absent up to 67 % of the time [2]. The risk of developing pneumonia has been found to be 4, 10, and 13 times greater, respectively, in patients with penetration, aspiration, or silent aspiration on videofluoroscopy compared to individuals with normal swallowing [3].

Evidence-based best-practice guidelines concur that screening protocols should be used to facilitate the prompt identification and management of aspiration risk in high-risk populations, such as stroke patients [4–7]. Screening for aspiration typically involves the swallowing of water. The clinician notes signs of difficulty, including cough, throat clearing, or voice changes that might imply the presence of liquid around the vocal cords. The utility of a screening tool, with respect to aspiration detection, should be measured in terms of its sensitivity (the % of participants who aspirate who are detected by the tool) and its specificity (the % of participants who do not aspirate who are correctly classified by the tool) [8]. Studies differ in their conclusions regarding the validity of abnormal clinical signs for revealing aspiration compared to blinded ratings of instrumental assessments [9–12]. As shown in Table 1, many swallow screening protocols have high false-positive rates, i.e., a tendency to over-identify aspiration. Notably, the instrumental assessments used for the purposes of validating screening results have typically been conducted separately from the screening procedure.

**Table 1:** Summary of previously reported sensitivity/specificity statistics for aspiration detection by swallow screening and clinical assessment tools compared to gold-standard instrumental swallowing examinations

Test and population	Population	Sensitivity (%)	Specificity (%)	False-positive rate (%)	False-negative rate (%)	Blinding?
Daniels swallow screen [8]	Acute stroke	92	66	33	8	Yes
Gugging swallow screen [9]	Stroke	100	50	50	0	Yes
Standardized clinical swallowing assessment [10]	Stroke	47	86	14	53	Yes
Volume-viscosity screening test [11]	Heterogeneous	100	29	72	0	Yes

Given the variable performance of swallow screenings for detecting aspiration, it would be desirable if a valid, noninvasive instrumental method, such as the appraisal of swallowing sounds or vibrations, could be developed to reliably detect aspiration at the bedside [13–15]. Unfortunately, perceptual clinical judgments of swallowing sounds do not lead to valid identification of aspiration, possibly because of a variety of artifacts that confuse perceptual analysis [16–19]. Signal processing may provide a means of overcoming these challenges, allowing for accurate detection of aspiration in physiological swallowing signals. Swallowing accelerometry is the study of swallowing vibrations measured on the neck and thought to arise from hyoid and laryngeal movement [20]. Research has shown that vibrations propagated in the anterior direction differ from those in the vertical axis [21]. Swallowing accelerometry signal duration has been shown to vary on the basis of body mass index, head position, age, and gender [19]. Certain signal characteristics are also known to vary across stimulus consistency [22]. Additionally, age, but not gender, influences the characteristics of cervical accelerometry signals at rest [18]. In the current study, we collected dual-axis cervical accelerometry signals from adults who completed a brief swallow screening protocol with concurrent videofluoroscopic observation in order to evaluate the performance of a novel aspiration-detection signal-processing classifier for detecting aspiration in these signals. Accelerometry-based classifier algorithms involving pattern recognition [23] have been successfully developed for other biomedical applications such as gait pattern analysis [24], falls detection [25], and dyskinesia assessment [26].

## Methods

### Participants

Participants included 40 adults (20 female; mean age = 67), referred for videofluoroscopy to investigate possible swallowing complaints. Table 2 provides additional details regarding the study sample breakdown by age and sex. Exclusion criteria included a known history of head and neck cancer, tracheostomy, neurodegenerative disease (including movement disorders), gastrointestinal disorders, or head and neck surgery (except routine tonsillectomy or adenoidectomy). Furthermore, all participants were required to be sufficiently alert to participate in a videofluoroscopic swallowing study and have adequate receptive communication and cognitive abilities to follow study instructions. Etiologies were not specifically tracked but were predominantly neurogenic. Some participants had no clearly identified medical condition that might explain their complaints of dysphagia. Current respiratory status (e.g., pneumonia, shortness of breath) was not considered in the study inclusion/exclusion criteria. There were no specified inclusion/exclusion criteria with respect to height, body mass index, or neck circumference given that a prior signal characterization study has shown limited influence of anthropometric parameters on signal features [27]. The study was approved by the institutional research ethics board.

**Table 2:** Participant demographics

Sex	No. of participants	Mean age	SD	Age range
Female	20	67	14	37–90
Male	20	67	14	40–90

### Data Collection

**Fig. 1:**
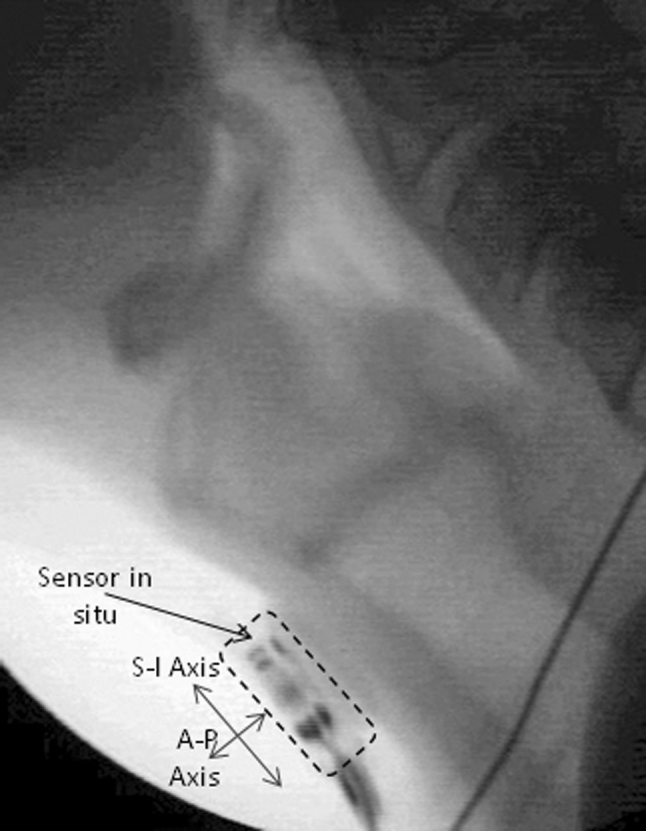
Videofluoroscopic image showing the accelerometry sensor in situ on the front of the neck, with the superior-inferior (S–I) axis aligned vertically with the surface of the participant’s neck and the anterior-posterior (A–P) axis derived at 90° to the S–I axis

Swallowing data were collected using a brief screening protocol of three 5-cc swallows and one cup-drinking task with Polibar powder for thin-liquid barium suspension (Bracco Imaging) diluted with water (40 g Polibar powder/250 ml water). The lateral view videofluoroscopy (VF) recording was captured and time-stamped at 30 frames per second in Labview software (National Instruments). Concurrent cervical accelerometry signals were collected via a dual-axis accelerometer (Analog Devices, ADXL322) attached to the participant’s neck in midline, anterior to the cricoid cartilage, using adhesive tape. Figure 1 shows the alignment of the sensor on a participant’s neck, with the superior-inferior (S–I) axis running vertically along the surface of the neck, and the anterior-posterior (A–P) axis derived at 90° to the S–I axis. The accelerometry axes were oriented so that the anterior and superior directions corresponded to positive signal polarities [20, 21]. The sensor was connected to the computer processing components of the data collection system via a single lightweight cable.

**Fig. 2:**
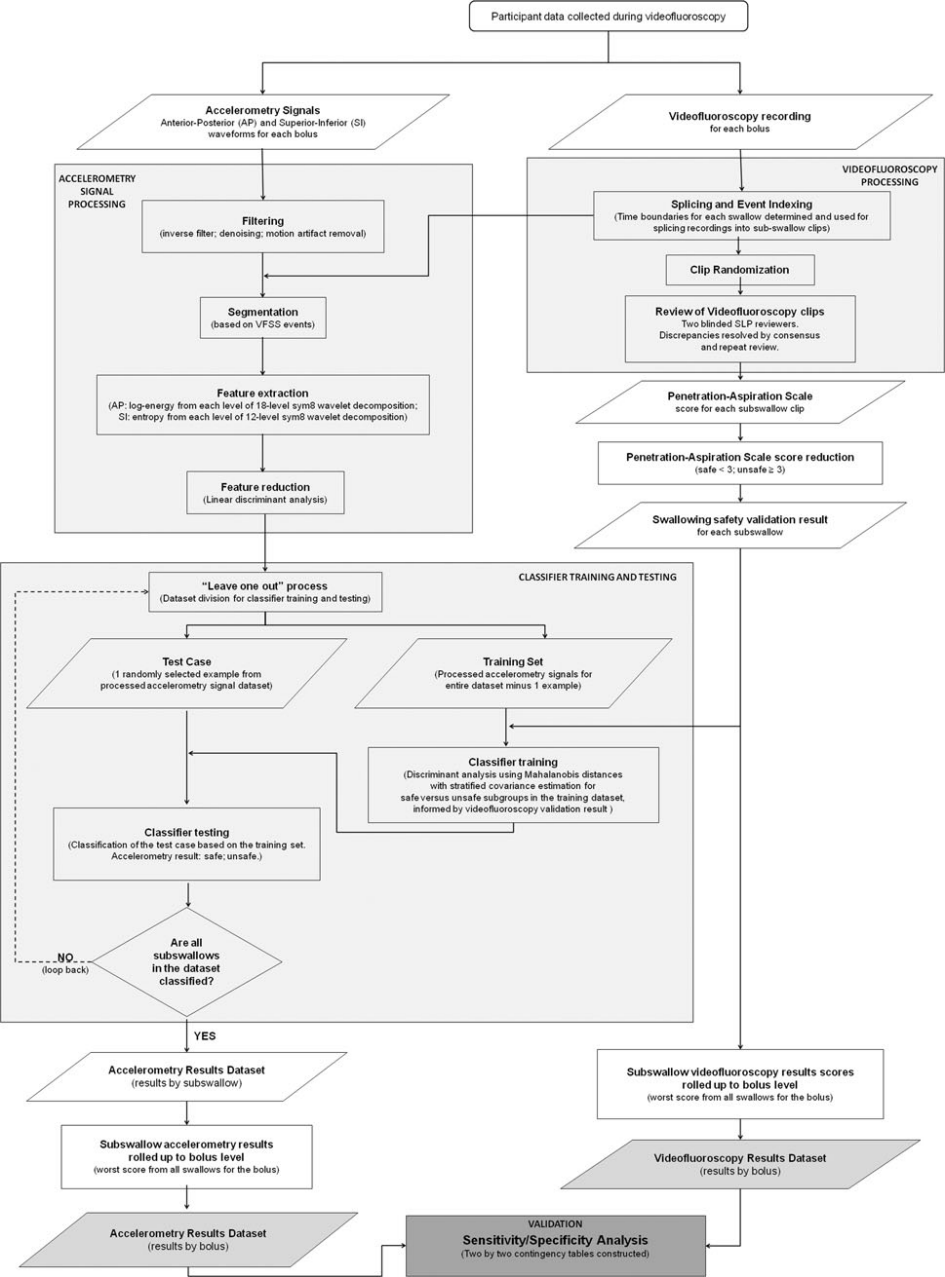
Flowchart showing the signal-processing steps used to analyze the dual-axis accelerometry signals

Figure 2 outlines the various data-processing and analysis steps of the study. These are described below.

### Videofluoroscopy Data Processing

The VF recordings were spliced into individual swallow clips capturing the interval between the arrival of the bolus head at the mandibular ramus and the lowest observed hyoid position following each swallow. Spontaneous clean-up swallows, following the initial swallow of each bolus, were spliced into separate clips, beginning at the lowest hyoid position before each new swallow event. These single-swallow clips were then arranged in random order and independently reviewed by two experienced speech-language pathologists, blinded to patient identity. The 8-point Penetration-Aspiration Scale [1] was used to rate airway invasion. Ratings were subsequently collapsed to a binary scale (≤2 vs. ≥3), distinguishing normal airway protection and high penetration (“safe”) from deeper entry of material into the airway without clearance (“unsafe”). Inter-rater agreement on initial ratings was good (intraclass coefficient = 0.81, 95 % confidence interval [CI] = 0.68-0.89) with a kappa score of 0.7 for binary results Disagreements were resolved by consensus through repeat review and discussion. The final set of binary results (safe; unsafe) comprised the data set of gold-standard “right answers” to which the accelerometry classifier signals were subsequently compared.

### Accelerometry Signal Processing

The processing steps applied to the accelerometry data were as follows. The signal was filtered and amplified (Astro-Med Inc., Grass, P55 A.C. preamplifier; bandpass-filtered 0.1 Hz–3 kHz; amplification 10×), then sampled at 10 kHz and stored on a computer with the time index corresponding to the videofluoroscopy timestamps. Inverse filters were then used for preprocessing [18], followed by denoising using a discrete Meyer wavelet transform with soft thresholding [19]. Previous studies have observed low-frequency components associated with head motion in swallowing accelerometry signals [21, 28]; therefore, we used a spline-based approach to remove these low-frequency components [29]. The signals were then manually segmented into swallow clips, based on the same timing boundaries used for segmentation and splicing of the VF recordings, as described above. From the segmented swallows, we extracted wavelet-based features of interest based on prior studies characterizing swallowing accelerometry signals [18, 21, 27].

### Classifier Algorithm Training and Testing

A leave-one-out approach with supervised classification [23] was used to categorize swallows as either safe or unsafe. For the purposes of this study, the entire set of processed accelerometry signals was divided into a training set (*N*–1) and a randomly selected test case. The gold-standard right answers regarding the binary presence or absence of Penetration-Aspiration Scale scores >3 from the blinded videofluoroscopy review were used to divide the training set into safe and unsafe categories, using discriminant analysis based on Mahalanobis distances and covariance estimates [30, 31]. Each test case was then classified using the trained algorithm, yielding a device result (safe; unsafe). The test case data were then added back into the data set and the process was repeated in an iterative fashion until all cases had been sampled and tested once and a complete set of device results was available.

### Validation

Validation of the classifier device results was then performed by comparing the device result with the gold-standard VF result on a swallow-by-swallow basis. Because some boluses were segmented into multiple swallows, both the device results and the VF results were rolled-up across all swallows for each single bolus to yield binary bolus-level results (problem; no problem), capturing the worst result across all subswallows for any given bolus. Thus, if a bolus sequence containing three subswallows showed a maximum Penetration-Aspiration Scale score of 2 across all three subswallows, it was scored as safe. Similarly, if one or more of three observed subswallows had a Penetration-Aspiration Scale score ≥3, the bolus sequence was scored as unsafe. Two-by-two contingency tables were constructed to allow the calculation of false-positive, false-negative, sensitivity, and specificity metrics.

## Results

Complete VF and accelerometry data were available for 37 participants. In the other three cases, image quality issues such as obstruction of the VF view of the airway by the shoulder shadow precluded verification of the classifier result. The final data set included 154 bolus swallow sequences, divided into 261 subswallows, with 31 % of these (80/261 swallows) displaying Penetration-Aspiration Scale scores of 3 or greater. Of the 154 bolus-swallowing sequences that were rated, 30 (19 %) displayed penetration-aspiration. When the data for all four swallowing tasks were considered in aggregate for each participant, penetration-aspiration was found to occur in 35 % (*n* = 13) of the 37 participants.

The results of the sensitivity/specificity analysis for penetration-aspiration detection by the accelerometry classifier are given in Table 3. Penetration-aspiration status was variable within participants such that the initial (i.e., first occurrence) episodes of penetration-aspiration for the 13 participants with impaired swallowing safety were distributed across the four thin-liquid swallowing tasks in the protocol. When the initial episode of penetration-aspiration occurred on the first teaspoon of thin liquid in the protocol, impaired airway protection was correctly identified by the classifier in all five cases. When the initial episodes of penetration-aspiration commenced with either the second (*n* = 3) or the third (*n* = 3) teaspoon of thin liquid, false-negative rates for the classifier were 34 % (sensitivity = 66 %) and 50 % (sensitivity = 50 %), respectively. The classifier showed no false negatives (i.e., 100 % sensitivity) in capturing the two episodes of penetration-aspiration that occurred for the first time during the final cup-drinking task.

**Table 3:** Accuracy statistics for the accelerometry signal-processing classifier algorithm for detecting aspiration in comparison to concurrent videofluoroscopy

Parameter	Statistic	Per bolus	Per participant
Impaired swallowing safety (13/37 patients; 55/154 swallows)	Sensitivity (%)	90	100
	Specificity (%)	77	54
	Negative predictive value (%)	97	100
	False-positive rate (%)	23	48
	False-negative rate (%)	10	0

## Discussion

In this study we recorded dual-axis cervical accelerometry signals during videofluoroscopy in adults suspected of having dysphagia. The signal-processing classifier trained in this study achieved false-negative rates of 10 % (90 % sensitivity) and false-positive rates of 23 % (77 % specificity) for detecting penetration-aspiration at the single-water-bolus level. These results compare favorably to those reported for other screening protocols, as summarized in Table 1. In a related analysis of this same data set [32], clinicians were asked to review concurrently captured movies showing the faces of these same participants performing the swallow screening tasks and to document any observed clinical signs of swallowing difficulty. The observation of abnormal clinical signs by registered nurses and speech-language pathologists, naïve to patient identity and clinical history, was found to have 54–75 % sensitivity for penetration-aspiration, but erred on the side of over-identification, with specificities of 25–44 %. The sensitivity and specificity metrics of these clinical perceptual judgments were similar to those reported by Leslie et al. [16] for perceptual judgments of 20 stethoscope-recorded swallowing sound clips by experienced speech-language pathologists (62 % sensitivity, 66 % specificity). Triangulation of the results across the two studies shows that our dual-axis cervical accelerometry signal-processing classifier performed better than perceptual judgments by clinicians for the very same swallows [32], with superior false-negative (0 %) and false-positive rates (46 %) for detecting penetration-aspiration across the aggregate of the four swallowing tasks observed for each participant. The signal-processing classifier had the added benefit of being able to drill results down to the bolus level, yielding false-negative rates of 10 % and false-positive rates of 23 %. Measures of classifier performance are expected to be higher at the resolution of the single bolus, given the fact that a protocol including several swallows provides a greater number of opportunities for a participant to demonstrate a single penetration-aspiration episode across several swallows. The decline in apparent accuracy at the level of the overall protocol simply reflects a difference in the resolution of the comparison, i.e., looking for at least one episode of penetration-aspiration over a denominator of four swallowing tasks versus a direct comparison at the level of a single bolus.

In this study, the protocol involved three teaspoon-sized boluses of thin liquid and a cup-drinking task. Other studies have suggested that the opportunity to catch aspiration (and sensitivity) increases with additional water swallow trials and have advocated for the inclusion of ten swallows in a screening protocol [33]. While we cannot speculate about the number of participants with no penetration-aspiration who might have displayed impaired swallowing safety given additional trials, our classifier correctly detected penetration-aspiration on either its first or second occurrence in all 13 participants who showed penetration-aspiration. In swallowing assessment, it is generally accepted that three repetitions of a task provide a representative sample of patient performance [34]. In this study, only 2 of the 13 participants with impaired swallowing safety showed their first episodes of penetration-aspiration on the final (i.e., fourth) cup-drinking task. These results support the use of the brief thin-liquid swallowing protocol used in this study as a valid and adequate method for identifying penetration-aspiration risk, but also point to the importance of including a larger-volume challenge, which may provoke penetration-aspiration in some patients who appear safe on smaller controlled volumes.

It is important to place the results of this study in context with other studies that have investigated the validity and utility of swallow screening protocols. Many of these prior studies have focused exclusively on patients with stroke [9–11, 35], and the phenomena of interest have ranged from aspiration below the true vocal folds (ignoring penetration, e.g., [10]) to a broader diagnosis of dysphagia, encompassing penetration, aspiration, and “any other abnormalities” of oropharyngeal swallowing physiology [35]. Clearly, when the definition of the target problem is set broadly, sensitivity for detection is likely to increase but specificity is likely to suffer. In some studies, participants who have been classified as failing swallow screening tests included individuals with a reduced level of consciousness that has precluded the inclusion of water-swallowing tasks [11]. In many cases, the results of a water-swallowing task have been compared to those of a subsequent (delayed) instrumental examination involving a variable number of tasks and stimuli [9, 11, 35]. This practice is in direct contrast to the procedures of the current study in which the screening result was validated in comparison to concurrent videofluoroscopy for the very same swallow. Where blinded instrumental ratings have been used to confirm the presence of aspiration in heterogeneous samples suspected of having dysphagia, high false-positive rates and poor specificity are findings of concern [12]. Specificity may be sacrificed to achieve high sensitivity in a screening procedure, but poor specificity can lead to the overzealous use of interventions that turn out to be unnecessary. With swallow screening, recognized aspiration risk provides a rationale for implementing severe dietary restrictions (e.g., nothing by mouth) until further assessment results are available. The accelerometry classifier used in this study showed 77 % specificity and 23 % false positives at the level of a single bolus, and 54 % specificity with 46 % false positives across the entire protocol of four thin-liquid swallowing tasks. These results still err on the side of over-identifying penetration-aspiration risk. Whether improved specificity, ruling out penetration-aspiration risk, could be achieved by altering the protocol (e.g., to include a larger-volume cup-drinking task at the end) is a question for future research.

Medical screening tests are intended, by definition, to be brief tests that yield a binary pass-fail result regarding a phenomenon of interest, in this case the occurrence of penetration-aspiration. As described by the World Health Organization, a screening test is not intended to be diagnostic or to provide sufficient information to guide management for those who exhibit the problem of interest [36]. In the case of swallow screening, the next step following a finding of concern should be referral for more comprehensive assessment, typically beginning with a clinical bedside swallowing examination (CBSA). The CBSA is a comprehensive noninstrumental assessment of oropharyngeal swallowing function and typically includes review of medical history, medication use, patient/caregiver reports of symptomatology, cognitive/behavioral factors that might impact swallowing, a detailed orofacial examination, voice assessment, and swallowing trials with a variety of stimuli [37]. The sensitivity and specificity for detecting aspiration by observing a cough, throat clearing, or wet voice following bolus swallows in such examinations have been reported as 81 and 47 %, respectively [38]. A more recent study that used the MASA (Mann Assessment of Swallowing Ability) in a general-medicine population at risk for aspiration reported sensitivity and specificity of 65 and 74 %, respectively, for the subjective ordinal risk rating score on that test [39]. Thus, the aspiration detection accuracy shown by our signal-processing classifier at the level of a single sip of water compares favorably to the accuracy of clinical screening results obtained using these other approaches. However, we note that a clinical swallowing assessment collects information that goes beyond an indication of aspiration risk; thus, such comparisons should be made with caution.

An important limitation of this study is that our data were collected from patients already suspected of having dysphagia to a degree where a videofluoroscopy had been ordered. As such, the probability of penetration-aspiration occurring is likely inflated compared to a nonreferred population. Future research will need to be done on samples where suspected dysphagia is not an inclusion criterion to confirm the sensitivity and specificity of the device classification algorithms for detecting penetration-aspiration in groups where swallow screening is recommended.

In conclusion, this study shows that dual-axis cervical accelerometry shows promise as a noninvasive tool for accurately detecting thin-liquid penetration-aspiration risk during a brief water swallow screening protocol. The signal-processing algorithms used in this study achieve penetration-aspiration detection rates that surpass clinical judges in accuracy, requiring only four swallowing tasks to reach a zero false-negative rate (100 % sensitivity). Specificity at the level of the single bolus was also strong, although the protocol and algorithms still erred on the side of caution with false-positive rates of 23 %. It is our opinion that a screening protocol involving the recording of dual-axis swallowing accelerometry signals, and automatic classification of those signals using the algorithms that were tested in this study could be implemented quite easily in early aspiration identification initiatives without requiring extensive nurse training, competency maintenance, and staffing resources. Such an approach holds promise to yield efficient, valid, and reliable indications of a patient’s aspiration risk without relying on the subjective interpretation of subtle clinical signs by nursing staff. We anticipate that the next steps in development of this tool will involve a large-scale device trial that compares thin-liquid screening results to videofluoroscopy. Larger samples of patients in specific etiological groups will be needed to demonstrate the utility of this device for use in swallow screening. It is our hope that this device will become commercialized in the future, facilitating prompt identification of aspiration risk and enabling the timely implementation of aspiration risk reduction strategies. Similarly, we hope that clinical uptake of this tool will reduce the number of patients who are unnecessarily placed on diet texture restrictions or referred for more invasive assessments, while prioritizing patients who require and stand to benefit from these more detailed assessments.
